# Dr. William Pepper

**Published:** 1898-09

**Authors:** 


### Obituary.

### DR. WILLIAM PEPPER.

Dr. William Pepper died July 28, 1898, of heart-disease, at the residence of Mrs. Phœbe Hearst, Pleasonton, California.

Dr. Pepper made this western trip on account of failing health, but neither family, friends, or professional advisers anticipated this early closing of his active life.

Dr. Pepper's father graduated with first honors at Princeton in the class of 1810, and became distinguished as the professor of the Theory and Practice of Medicine in the University of Pennsylvania. The son graduated from the Collegiate Department of this university in 1862, and subsequently from the Medical Department.

He became connected with the teaching department of his Alma Mater in 1868, and for two years thereafter was a lecturer on morbid anatomy before the medical classes. He was then transferred to the Department of Clinical Medicine, and, after six years as lecturer on that subject, was elected to the full professorship, and held the chair until 1884. In the last named year he succeeded Dr. Alfred Stillé as professor of the Theory and Practice of Medicine, a chair which he held down to the time of his death.

Meanwhile, in January, 1881, Dr. Pepper was unanimously elected provost of the university, in which office he succeeded Dr. Charles J. Stillé. This responsible position he filled for thirteen years, retiring from it in June, 1894. Under his administration the growth of the university was more marked than at any previous period in its history. Dr. Pepper was a man of extraordinary executive ability, of restless activity, and of the broadest, most liberal, and most comprehensive views on the general subject of education. Having inherited a large fortune, he was enabled to enforce his progressive ideas by liberal donations to the institution in whose welfare he took such an earnest interest and such an active part. His most important work, perhaps, in the development of the university was that accomplished by him in the direction of higher medical education. This advance was secured through the extension of the course to four years, and was stimulated by the following proposition, which he made in 1891:

"Being impressed with the urgent importance of higher medical education, and believing that it is the duty and the privilege of the Medical Department of the University of Pennsylvania to be the leader in this the final reform of medical education in America, I beg to submit the following proposition: That on condition the university shall decide and announce before June 1, 1891, that a four-year obligatory graded course of medical study shall be established on or before September 1, 1891, I will subscribe towards a permanent endowment fund of $250,000; for the Medical Department the sum of $50,000, subject to the conditions below recited; and towards a guarantee fund of $20,000 per annum for five years, the sum of $1000 annually during that period."

The report which Dr. Pepper submitted to the board of trustees at the close of his administration as provost, and covering the period of the thirteen years of its continuance, gave a clear idea of the growth of the institution. This report presented the following comparative figures:

| | 1881. | 1894. |
|---|---|---|
| Land . . . . . . . . . . . . . . . . | 15 acres. | 52 acres. |
| Number of students . . . . . . . . . | 991 | 2180 |
| Fees of students . . . . . . . . . . . | $92,701 | $230,567 |

The report further showed that during Dr. Pepper's term as provost the following departments were created: The Wharton School, Veterinary School and Hospital, the Biological School, the Graduate Department, Department for Women, the Department of Physical Education, the Department of Archæology, and the Laboratories of Hygiene and of Chemistry. The medical school course had been extended to four years, and those of the dental and law to three each, elective and technical courses had been developed in the college, and important auxiliaries, such as the Lecture Association, the Archæological Association, and the American Society for University Extension created. The growth of the University Hospital during the same period was also very great, culminating, as it did, in the erection of the D. Haynes Agnew Memorial wing, the Maternity Hospital, and the William Pepper Laboratory of Clinical Medicine, Provost Pepper's own memorial to his father.

In addition to his duties at the university and with the practice of his profession Dr. Pepper has regularly continued his literary work. He founded the *Medical Times*, and was its editor in 1870–71. His most important literary work has been the editing of the "System of Medicine by American Authors" (five volumes, Philadelphia, 1885–86). This secured an immediate success, and was recognized as the chief American authority on medical questions. He published, in conjunction with Dr. John F. Meigs, successive editions of their work on "Diseases of Children" (1870). He was also a frequent contributor to the leading medical journals and the transactions of the medical societies. Among other publications from his pen have been his inaugural address and annual reports as provost, and public addresses on "Force *vs.* Work" (Baltimore, 1884), and "Benjamin Franklin" (Lancaster, 1887).

He was medical director of the Centennial Exhibition in 1876, and for his services therewith received from the king of Sweden the decoration of the Knight Commander of the Order of St. Olaf.

He was a member of the various learned societies of Philadelphia, and was elected president of the first Pan-American Medical Congress, held at Washington in 1893. In 1881 he received the degree of LL.D. from Lafayette College, and the same honor from Princeton in 1888. He was the principal originator of the Commercial Museums of Philadelphia, and subsequently became president of this important and justly celebrated enterprise. It was mainly through his efforts that the Free Library of Philadelphia was established upon such a broad, liberal basis that it is made available throughout the entire city.

His work as provost of the University of Pennsylvania exhibits, perhaps, more completely the character of the man, and will, doubtless, bring to him the most enduring honor. Its marvellous growth has been previously outlined, but figures can never tell of the exhausting labor given by him to effect the results stated. His aim was not to fill the grounds with buildings, important as he regarded these, for his conception of a university existed not in stone, bricks, and mortar, but in men, and therefore it was his ambition to have these structures occupied with the highest possible culture attainable. The result was soon manifest in every department, and the commanding position attained by the university under his leadership will be continued by his successor, Provost Harrison.

His interest in the Department of Dentistry never lessened. He once stated to the writer that in his earlier years he felt a strong prejudice against dentistry, which had entirely been removed through a closer intercourse with men connected with it and an increased knowledge of its value.

To the writer he was not only his superior in office, but a personal friend, ever ready to aid in any movement for the improvement of the Department of Dentistry, and to his earnest co-operation must be ascribed the large measure of success achieved.

While Dr. Pepper's fame will rest mainly upon his medical work, dentistry will ever remain indebted to him for his cordial co-operation in the efforts put forth to raise its standard. He was closely in touch with the National Association of Dental Faculties, and at once had it recognized by the authorities of the university and its regulations accepted as the guide for the Department of Dentistry.

To the writer his death leaves a vacancy among the list of those he honored, and the world seems all the more lonely that this active, brilliant mind is no longer a worker in it. While the majority of men labor to exalt their individual ambitions, Dr. Pepper unselfishly toiled to raise the standard of happiness, to increase human knowledge, and to leave the world better than he found it. Who will deny him the honor of having more than accomplished his ambitions?

In 1873, Dr. Pepper was married to Miss Florence Sergeant Perry, a granddaughter of Commodore Oliver Hazard Perry, and a lineal descendant of Benjamin Franklin. Four sons were born to Dr. and Mrs. Pepper, three of whom survive. These are Dr. William Pepper, Jr., who has been assisting his father; B. Franklin Pepper, a private in Battery A; and Oliver Hazard Perry Pepper, still at school.

